# Determinants of HIV-malaria co-infection among people living with HIV on anti-retroviral therapy in Northeast Ethiopia: unmatched case control study

**DOI:** 10.1186/s41182-020-00286-9

**Published:** 2020-11-30

**Authors:** Tenaw Yibeltal, Dereje Birhanu Abitew, Amsalu Birara Melese, Yared Mulu

**Affiliations:** 1 Department of Epidemiology and Biostatistics, School of Public Health, College of Medicine and Health Science, Bahir Dar University, P.O. Box 79, Bahir Dar, Ethiopia; 2 Department of Public Health Nutrition, School of Public Health, College of Medicine and Health Science, Bahir Dar University, P.O. Box 79, Bahir Dar, Ethiopia; 3 Department of Environmental Health, School of Public Health, College of Medicine and Health Science, Bahir Dar University, P.O. Box 79, Bahir Dar, Ethiopia; 4 Department of Health System and Health Economics, School of Public Health, College of Medicine and Health Science, Bahir Dar University, P.O. Box 79, Bahir Dar, Ethiopia

**Keywords:** Malaria, HIV/AIDS, Co-infections, PLWHA

## Abstract

**Background:**

HIV and malaria are the leading causes of morbidity and mortality in the developing world including Ethiopia. Globally, HIV-malaria co-infection causes approximately 3 million deaths per year. However, both these infections are preventable if measures are taken on determinant factors. The objective of the study was therefore to assess factors associated with HIV-malaria co-infection among HIV-positive people who lived in Shewarobit district, northeast Ethiopia.

**Methods:**

Unmatched case-control study was conducted among people living with HIV (PLWHA) in Shewarobit district from February 28, 2018, to April 30, 2018. The sample size was determined taking the assumption of 95% CI, 85% power, 3:1 control to case ratio, the proportion of PLWHA-malaria coinfection of 22.7%, OR 2.73, and 10% non-response rate. The final sample size was 262 (66 cases and 196 controls). Cases were adults on anti-retroviral therapy and diagnosed positive for malaria by microscopy while controls were adults on anti-retroviral therapy and diagnosed negative for malaria by microscopy in the previous 6 months before the survey.

**Result:**

The median age of cases and controls in years was 35 (IQR = 19) and 38 (IQR = 19) respectively. Variables that had a significant association with HIV-malaria co-infection were non-in-door residual spraying (adjusted odds ratio (AOR) = 4.91; 95% CI 4.03, 15.13), poor perception on the health risk of HIV-malaria co-infections (AOR = 4.11; 95% CI 1.28, 10.17), non-use of insecticidal treated bed nets (AOR = 6.21; 95%CI 2.74, 14.11), non-use of cotrimoxazole prophylaxis (AOR = 2.42; 95% CI 1.11, 5.28), and not received health education on the risk of HIV-malaria interaction (AOR = 4.11; 95% CI 1.24, 4.84).

**Conclusion:**

Provision of cotrimoxazole prophylaxis, sleeping under an insecticidal treated bed net, and indoor residual spraying help to reduce HIV-malaria co-infection-associated morbidity/mortality.

## Introduction

### Background

Co-infection is the concurrent infection of a host by multiple pathogenic species [1] and has serious health consequences than a single infection [2]. Malaria and HIV are the two most important global health problems that have similar geographical distribution and are the leading causes of morbidity and mortality, predominantly in sub-Saharan Africa [3, 4] and malaria is a life-threatening parasitic disease caused by a single-celled protozoon. Of the five *Plasmodium* species (*P*. *falciparum*, *P*. *vivax*, *P*. *ovale*, *P*. *malariae*, and *P*. *knowlesi*), only four of the species (*P*. *falciparum*, *P*. *vivax*, *P*. *ovale*, and *P*. *malariae*) cause malaria infection to humans. *Plasmodium falciparum* and *P*. *vivax* cause most of the morbidity and mortality worldwide and then, *P*. *falciparum* is the most dangerous *Plasmodium* species in terms of disease severity and is the most prevalent parasite in Africa, while *P*. *vivax* is less severe but more widely distributed in several countries outside of Africa [5–7].

The clinical symptoms of malaria are “fever or fever with a headache, back pain, chills, rigor, sweating, muscle pain, nausea, and vomiting.” The most typical symptoms of malaria are “the periodic attacks of fever” and “the malarial rigor.” Low body temperatures or hypothermia may be seen in young children [8]. The incubation period of the disease varies from species to species where it is 5–7 days for *P*. *falciparum*, 6–8 days for *P*. *vivax* 6–8 days, 8–9 days for *P*. *ovale*, and 12–16 days for *P*. *malariae* [5].

Malaria is transmitted by the female *Anopheles* mosquito. There are 400 species in the world. However, only about 30 species transmit malaria to humans. The mosquito distribution varies from country to country. The type of mosquito that transmits malaria in Ethiopia is *Anopheles arabiensis* [7]. On the other hand, human immunodeficiency virus (HIV) is a retrovirus that infects and then deteriorates human immune cells resulting in the inability to fight against infections [9]. HIV is transmitted primarily by unprotected sex (including anal and oral), contaminated blood transfusions, sharing contaminated sharp materials, and from mother to child during pregnancy, delivery, or breastfeeding [9]. HIV has no radical cure. However, highly active anti-retroviral therapy (HAART) can reverse and improve clinical status in keeping with immune recovery and suppression of viral load. The CD4 count usually increases in response to effective combination anti-retroviral therapy [10].

Some population groups are at higher risk of contracting malaria and developing a severe form of the disease than others. These include pregnant women, infants, children under 5 years, patients with HIV/AIDS, non-immune migrants, mobile populations, and travelers [9, 11]. Malaria and HIV/AIDS disproportionately affect developing countries [12].

The coinfection of HIV-malaria has effects on an individual’s health and nutrition status which therefore could put at risk of morbidity and mortality. Studies in Nigeria and Ethiopia indicated that patients with dual infection of malaria and HIV were more anemic than their counters [13, 14]. People with HIV-malaria co-infection were reported to have low immunity and reduces CD4 count [15], and the risk of death in PLHA coinfected with malaria is also reported high due to low anti-retroviral therapy (ART) treatment adherence [16]. Coinfection also leads to adverse birth outcomes (abortion, pre-term delivery, and low infant birth weights) as reported in Nigeria [17].

Even though both HIV and malaria are important public health problems and both of these infections are preventable if measures are taken on determinant factors [18]. The factors associated with HIV-malaria co-infection were not identified in the study area. The objective of the study was therefore to provide information on the factors associated with malaria infection among people living with HIV/AIDS (PLWHA) attending a Shewarobit health center. The study could help program managers/implementers to assess/evaluate whether HIV-malaria prevention and control activities are practiced as stated in the guideline to protect HIV-positive individuals from malaria infection.

## Method

### Study area and setting

The study was conducted in the Shewarobit district of North Showa Zone, Amhara region, Ethiopia. Shewarobit is one of the 24 districts in the North Shewa zone, Amhara Region, Ethiopia. It is located 225 km to the North of Addis Ababa. The district has a longitude and latitude of 10° 00′ N 39° 54′ E with an elevation of 1280 m above sea level. The district has 9 kebele (4 urban and 5 rural). Based on projections from the 2007 Ethiopian national census, the total population of Shewarobit district in 2018 was 45,528 of which 23,219 were females. During the study period, the district has a total of three health facilities (one health center, one governmental hospital, and one private hospital) but only Shewarobit Health center was providing ART service as the government hospital was the newly constructed hospital and the private was not providing ART service. According to North Shewa Zone health management and information system (HMIS) report, a total of 1362 PLWHA were on ART at Shewarobit health center.

### Study design

An unmatched case-control study was conducted from February 28 to April 30, 2018.

### Source and study population

The source population for the cases was those who were on ART and positive for malaria microscopic result (at least once) on their medical record, while the study populations were people over 18 years of age who were on ART and positive for malaria microscopic result (at least once) on their medical record in the previous 6 months before the start of the study.

The source population for the controls was those who were on ART and negative for malaria microscopic result (at least once) on their medical record, while the study population was people over 18 years of age who were on ART and negative for malaria microscopic result (at least once) on their medical record in the previous 6 months before the start of the study.

### Sample size determination and sampling techniques

The sample size was computed using Epi info version 7 statistical software considering the following assumptions: 95% CI, power 85%, 3:1 a ratio of controls to cases, and the proportion of HIV-malaria coinfection among PLWHA living in an area for less than 5 years (non-exposed to the malaria of 22.7%), the odds ratio of 2.73 [19], and then adding 10% non-response rate. The total sample size was 262 (66 cases and 196 controls). Client’s medical record was reviewed and those with at least one positive malaria microscopic test result in the previous 6 months were listed and also those clients who were negative for malaria were listed separately. Therefore, using the list as a sampling frame, cases (198) and controls (1164) were selected using a systematic random sampling technique with a sampling interval of 6 for controls and 3 for cases.

### Data collection tools and techniques

A checklist and a structured and pretested interviewer-administered questionnaire were used to collect data. Medical records were retrieved using the checklist and the interview technique was face-to-face. The data was collected by four trained clinical nurses who were fluent speakers of the local language (Amharic) in a quiet place (room) in the anti-retroviral therapy clinic to increase participant’s confidentiality and ensure privacy. One senior nurse professional and the principal investigators were responsible for the overall supervision of the data collection process.

### Operational definitions

#### Case

Adult people living with HIV who were on ART and positive for malaria microscopic result (at least once) on their medical record in the previous 6 months before the start of the study.

#### Controls

People living with HIV who were on ART and negative for malaria microscopic test result on their medical records in the previous 6 months before the start of the study

#### ITN use

People who were reported to have slept under an insecticide-treated bed net (ITN) the night before the date of the interview were considered ITN users.

#### In-door residual sprayed house

A house that reported being insecticide residual sprayed (IRS) within the previous 6 months before the survey

#### Cotrimoxazole (CTX) user

Those who reported on cotrimoxazole prophylaxis daily and documented on their medical chart were considered CTX users.

#### Good perception of risk of HIV-malaria co-infection

A respondent was considered to have a good perception of the risk of HIV-malaria co-infection if he/she correctly answers three or more of the five risk perception questions.

### Data quality assurance

The original English version of the questionnaire was translated first to Amharic (the local language) and back to English by the individual who has good knowledge both in English and Amharic languages to assure its consistency. The pretest was conducted in another health center. The one-day training was provided for data collectors and supervisors. The supervisor was checking the collected data for completeness on daily basis and the uncompleted questionnaire was returned to the data collector for correction to maintain its accuracy and completeness and also spot-checking by principal investigators.

### Data processing and analysis

The collected data were checked manually for completeness, consistency, and accuracy before analysis. Data were coded, cleaned, and entered into Epi-Infoversion7 then exported to SPSS v20 for analysis. Both bivariable and multivariable binary logistic regression analyses were used to identify associated factors. Variables having a *p* value of less than 0.2 in the bivariable analysis were entered into the multivariable model to control the effect of confounding. Hosmer-Lemeshow goodness-of-fit statistics were used to assess the fitness of the model. The result was presented by text and table. Variables with a *p* value less than 0.05 were considered significant determinants of HIV-malaria coinfection and odds ratios with 95 % CI were calculated to measure the strength of association.

## Results

### Socio-demographic characteristics of respondents

A total of 244 (61 cases and 183 controls) participants were included in our study with a 91.7% response rate. Of the total study participants, 142 were females. The median age of cases and controls in years was 35 (IQR = 19) and 38 (IQR = 19), respectively. Twenty-seven (44.3%) cases and 83 (45.4%) controls were unable to read and write. A relatively higher percentage of cases (41.0%) had a monthly income of less than 600 ETB as compared to controls (29.5%) (Table 1).

**Table 1:** Socio-demographic characteristics of PLWHIV in Shewarobit Health Center, Shewarobit district, Amhara Region, Ethiopia, 2018

Variables	Categories	Cases (# (%))	Controls (# (%))
Age in years	19–24	13 (21.3)	20 (10.9)
	25–29	10 (16.4)	31 (16.4)
	30–39	16 (26.2)	54 (29.5)
	40+	22 (47.5)	78 (42.6)
Sex	Male	29 (47.5)	73 (39.9)
	Female	32 (52.5)	110 (60.1)
Educational status	Unable to read and write	27 (44.3)	83 (45.4)
	Primary (grade 1–8)	18 (29.5)	64 (34.9)
	Secondary (grade 9–12)	8 (13.1)	24 (13.1)
	College and above	8 (13.1)	12 (6.6)
Window(s) condition	Covered window	19 (31.1)	81 (44.3)
	Uncovered window	24 (39.3)	57 (31.1)
	No window	18 (29.5)	45 (24.6)
Hole(s) wall of the house	Yes	16 (26.2)	20 (10.9)
	No	45 (73.8)	163 (89.1)
Occupation	Farmer	19 (31.2)	49 (26.8)
	Small trade/business	16 (26.2)	72 (39.3)
	Employed (govt/private)	13 (21.3)	29 (15.8)
	Daily laborer	7 (11.5)	18 (9.8)
	Others (student/jobless)	6 (9.8)	15 (8.2)
Current marital status	Married	42 (68.9)	145 (79.2)
	Not married	19 (31.2)	38 (20.8)
Residence	Rural	22 (36.1)	64 (35.0)
	Urban	39 (63.9)	119 (65.0)
Length of stay in Shewarobit	< 5 years	7 (11.5)	18 (9.8)
	≥ 5 years	54 (88.5)	165 (90.2)
Monthly income in ETB	≤ 600	25 (41.0)	54 (29.5)
	601–900	23 (37.7)	77 (42.1)
	901–1399	7 (11.5)	27 (14.8)
	≥ 1400	18 (29.5)	25 (13.7)
Health education on HIV-malaria interaction	Yes	13 (21.3)	73 (39.9)
	No	48 (78.7)	110 (60.1)
Good perception of risk of HIV-malaria co-infections	Yes	21 (34.0)	123 (67.0)
	No	40 (66.0)	60 (33.0)
Daily CTX use	Yes	19 (31.2)	122 (66.7)
	No	42 (68.9)	61 (33.3)
IRS within the past 6 month	Yes	28 (45.9)	159 (86.9)
	No	33 (54.1)	24 (31.1)
ITN used	Yes	19 (31.1)	141 (77.1)
	No	42 (68.9)	42 (23.0)

*HE* health education, *CTX* cotrimoxazole, *ETB* Ethiopian Birr, *IRS* insecticide residual sprayed

### Determinants of HIV-malaria co-infection among people living with HIV on ART in Shewarobit district, northeast Ethiopia

Bivariable logistic regression analysis was used to identify candidate variables and nine variables (marital status, occupation, in-door residual spraying (IRS), perception about HIV-malaria co-infections, ITN use, hole(s) on the wall of houses, health education on HIV-malaria interaction, houses window(s) condition, use of cotrimoxazole prophylaxis) were with *p* value < 0.2 and were entered into the final model. On multivariable logistic regression analysis, five variables (in-door residual spraying (IRS), perception about HIV-malaria co-infections, health education on HIV-malaria interaction, ITN use, and cotrimoxazole use) remain independently associated with HIV-malaria co-infection.

Participants who reported their house was not sprayed (IRS) were five times more likely to have HIV-malaria co-infectious than those whose house was sprayed (adjusted odds ratio (AOR) = 4.91; 95% CI 2.14, 11.23), and also participants who did not use ITN daily were 6.21 times more likely for HIV-malaria co-infection than those who used ITN (AOR = 6.21; 95% CI 2.74, 14.11; *p* < 0.001). Moreover, participants who have not received health education on the health risk of HIV malaria interaction were 4 times more likely to develop HIV-malaria co-infected than their counterparts (AOR = 4.11; 95% CI 1.65, 10.17; p = 0.002) (Table 2).

**Table 2:** Bivariable logistic regression analysis of determinant factors for HIV-malaria co-infection among people living with HIV attending Shewarobit Health Center ART Clinic, Shewarobit district, Amhara Region, Ethiopia 2018

Variables	Categories	Cases	Controls	95% CI	
		# (%)	# (%)	COR	AOR
Current marital status	Married	42 (68.9)	145 (79.2)	1	1
	Not married	19 (31.2)	38 (20.8)	1.73 [0.90, 3.30]	2.04 [0.85, 4.90]
Occupation	Farmer	19 (31.2)	49 (26.8)	1	1
	Trader	16 (26.2)	72 (39.3)	0.57 [0.22, 1.22]	0.65 [0.17, 2.42]
	Employer	13 (21.3)	44 (16.9)	1.08 [0.46, 2.49]	2.11 [0.49, 8.99]
	Others	13 (21.3)	44 (16.9)	1.08 [0.46, 2.49]	1.10 [0.29, 4.13]
HH window(s) condition	Covered	19 (31.1)	81 (44.3)	1	1
	Uncovered	24 (39.3)	57 (31.1)	1.79 [0. 90, 3.58]	1.38 [0.47, 4.05]
	No window	18 (29.5)	45 (24.6)	1.7 [0.81, 3.57]	1.04 [0.25, 3.98]
Hole(s) on the wall of a house	Yes	16 (26.2)	20 (10.92)	1	1
	No	45 (73.8)	163 (89.1)	0.34 [0.16, 0.72]*	0.82 [0.31, 2.17]
HE on HIV-malaria interaction	Yes	13 (21.3)	73 (39.9)	1	1
	No	48 (78.7)	110 (60.1)	2.45 [1.24, 4.84]*	4.11 [1.65, 10.17]*
Good perception of HIV-malaria co-infections	Yes	21 (34)	123 (67.0)	1	1
	No	40 (66)	60 (33.)	3.9 [2.12, 7.19]*	4.11 [1.28, 10.17]*
Daily CTX use	Yes	19 (31.2)	122 (66.7)	1	1
	No	42 (68.9)	61 (33.33)	4.42 [2.37, 8.24]*	2.42 [1.11, 5.28]*
IRS of house	Yes	28 (45.9)	159 (86.9)	1	1
	No	33 (54.1)	24 (31.1)	7.8 [4.03, 15.13]*	4.91 [2.14, 11.23]*
ITN use	Yes	19 (31.1)	141 (77.1)	1	1
	No	42 (68.9)	42 (23.0)	7.42 [3.90, 14.10]*	6.21 [2.74, 14.11]*

*HH* household, *HE* health education, *CTX* cotrimoxazole, *IRS* insecticide residual spraying, *ITN* insecticide-treated net

*P< 0.05

## Discussion

In the current study, an attempt was made to identify factors associated with HIV malaria co-infection among those who were on ART. From the candidate variables included in the final model, daily use of cotrimoxazole as prophylaxis, consistent use of ITN at bedtime, and spraying households with residual insecticide were found less at risk for malaria infection. Moreover, those PLWHA who were aware of the risk of being infected with malaria and those who reported got health education on the interaction of HIV and malaria on their health were less affected by the coinfection of HIV and malaria.

The WHO recommends the continuation of cotrimoxazole prophylaxis regardless of CD4 cell count or WHO clinical stages in settings where malaria is highly prevalent [20]. In our study, those who did not take CTX prophylaxis were found more infected with malaria than those who take cotrimoxazole prophylaxis daily. The finding was consistent with the study conducted in Uganda which reported that study participants on ART who stopped taking CTX prophylaxis were more likely to be infected by malaria than those who continued taking CTX prophylaxis [21]. Similarly, studies in the Northern part of Ethiopia and Oromia region Ethiopia indicated that PLWHA who were taking cotrimoxazole prophylaxis were more protected against malaria infection than PLWHA who were not taking cotrimoxazole prophylaxis [22, 23]. Possible explanation CXT can recover the immunity system by preventing and treating malaria and severe bacterial infections in adults and children taking ART [24].

Studies reported that integration of ITN use with the provision of ART and CTX prophylaxis reduced malaria incidence among HIV-positive people by 95% [21, 25]. In our study, those who reported not used ITN daily were found more infected with malaria compared to those who used ITN daily. The finding was consistent with a systematic review conducted in low- and middle-income countries in that, 3 months after the provision of ITN to PLWHA, the risk of malaria infection was highly reduced [26]. A post-intervention evaluation on the effect of the long-lasting insecticide-treated net (LLITN) on the incidence of malaria among PLWHA in Nigeria showed that the incidence of malaria was reduced from 35% to 6.0% 6 months after intervention [27]. Similarly, studies in the Northern and the Oromia region, Ethiopia, reported that PLWHA who used ITN were less at risk of malaria infection [22, 23]. Possible explanation could be because, insecticide-treated bed net kills a mosquito, reduce numbers by its repelling effect, and also as physical barriers [28].

In contrast, a study in Dembia district, Northwest Ethiopia, reported that bed net utilization had no statistically significant association with malaria infection [29]. The difference might be due to vector resistance or the condition of bed nets.

Provision of health education to PLWHA on HIV and malaria interaction is a vital tool for improving their knowledge so that malaria prevention activities will be effective [30]. In the current study, those who reported not received health education about the risk of HIV malaria coinfection and also those who did not perceive the risk of being infected with malaria were more at risk for malaria infection than their counterparts. The finding was in line with a study conducted in Indonesia that PLWH with poor health knowledge was more at risk of recurrence in PLWH co-infected with malaria than their counters [16]

The possible reason for this might be because those who got such an education regarding the health risk of malaria infection might have perceived its deadly effect, so they could take preventive measures such as proper ITN use. This is exemplified by the fact that in the randomized controlled trial in Nigeria the use of ITN and environment management practices were improved in the intervention than in control groups. The proportion of ITN users among intervention groups increased from 50.8 to 87.4% whereas the proportion of ITN users in the nonintervention group increased from 40.4 to 54.5% [31].

As part of the malaria eradication program, IRS has been implemented since 1955 in several countries like Eritrea, Ethiopia, and Madagascar where malaria is highly seasonal [32]. According to our findings, respondents who reported that their house was not sprayed recently were more at risk of being infected with malaria. The finding was in agreement with a study conducted in Western Kenya that a recent history of fever was more reported among PLWHA in the non-IRS districts than those in the IRS district [33]. This might be that people living in the districts which have IRS program had a higher chance of spraying their houses than those districts which have not IRS program. Another study in Malawi indicated that malaria prevalence among people living in sprayed houses was lower by 33% as compared to people living in non-sprayed houses [34]. However, the study in Demba district reported that IRS was not statistically associated with malaria infection [29] maybe because the mosquitoes in Demba district might have developed resistance to the type of chemical sprayed in the area.

### Limitation of the study

We did not use observation as a data collection tool and ITN daily use, household IRS, and housing conditions were based on the respondent’s report. Respondents might also have recall bias in some of the issues raised. Household income was estimated merely by asking respondents and not based on the standard income estimation technique.

## Conclusion

Our study identified that the daily use of CTX prophylaxis, proper use of ITN, educating PLWHA on the health risk of HIV-malaria coinfection, and the IRS of the houses were the important determinants to reduce the risk of HIV-malaria co-infection. Therefore, health program managers/implementers need to evaluate whether HIV-malaria prevention and control activities are integrated along with an ongoing awareness creation to PLWHA on the health risk of coinfection with malaria and biannually IRS of houses need to be implemented to reduce HIV-malaria coinfection-related morbidity and mortality. We also recommend further study using follow-up design to identify more morbidity reports and other variables not included in this study.

## Data Availability

The datasets used and/or analyzed during the current study are available from the corresponding author on request.

## Abbreviations

AOR: Adjusted odds ratio
ART: Anti-retroviral therapy
AIDS: Acquired immune deficiency syndrome
CTX: Cotrimoxazole
ETB: Ethiopian Birr
HAART: Highly active anti-retroviral therapy
HIV: Human immunodeficiency virus
WHO: World Health Organization
HMIS: Health management information system
PLWHA: People living with HIV/AIDS
ITN: Insecticidal treated net
LLITN: Long-lasting insecticidal treated bed net

## References

[1] Platt L, Easterbrook P, Gower E, McDonald B, Sabin K, McGowan C (2016). Prevalence and burden of HCV co-infection in people living with HIV: a global systematic review and meta-analysis. Lancet Infect Dis.

[2] Griffiths EC, Pedersen AB, Fenton A, Petchey OL (2011). The nature and consequences of coinfection in humans. J Infect.

[3] Malaria RB (2005). World malaria report 2005. World Health Organization and UNICEF.

[4] WHO (2005). High risk groups for malaria.

[5] WHO, UNICEF (2000). Achieving the malaria MDG target, reversing the incidence of malaria 2000–2015.

[6] Hawker J, Begg N, Blair I, Reintjes R, Weinberg J. Communicable disease control handbook. Wiley; 2008.

[7] World Health Organization. Malaria elimination: a field manual for low and moderate endemic countries. Malaria elimination: a field manual for low and moderate endemic countries. 2007.

[8] Ethiopia: FDRo (2012). Public health emergency management guidelines for Ethiopia.

[9] WHO (2017). World malaria report, 2017.

[10] WHO (2007). Strengthening health services to fight HIV/AIDS.

[11] UNAIDS,GLOBAL STATISTICS,HIV/AIDS FACT SHEET. 2014. http://www.aids2014.org/webcontent/file/AIDS2014_Global_Factsheet_April_2014.pdf.

[12] World Vision, The Link between Malaria and HIV and AIDS, Roll Back Malaria. https://www.wvi.org/sites/default/files/HIV_and_Malaria_flyer_English.pdf.

[13] Sanyaolu AO, Fagbenro-Beyioku A, Oyibo W, Badaru O, Onyeabor O, Nnaemeka C (2013). Malaria and HIV co-infection and their effect on haemoglobin levels from three healthcare institutions in Lagos, southwest Nigeria. Afr Health Sci.

[14] Wondimeneh Y, Ferede G, Atnafu A, Muluye D (2013). HIV-malaria co-infection and their immunohematological profiles. Eur J Exp Biol.

[15] Jegede FE, Oyeyi TI, Abdulrahman SA, Mbah HA, Badru T, Agbakwuru C (2017). Effect of HIV and malaria parasites co-infection on immune-hematological profiles among patients attending anti-retroviral treatment (ART) clinic in Infectious Disease Hospital Kano, Nigeria. PLoS One.

[16] Winiarti D, Mudigdo A, Murti B (2019). Determinants of recurrence and death in HIV-malaria co-infection patients in Jayapura, Papua, Indonesia. J Epidemiol Public Health.

[17] Dibua UM, Badger-Emeka L, Ugonabo JA (2013). HIV and malaria co-infection: their combined effects on pregnancy outcomes in Anambra state, southeast Nigeria. Int J Med Med Sci.

[18] WHO (2017). WHO world malaria report,2017.

[19] Inchana W, Kamchoo K, Wetasin K (2013). Factors associated with malaria infection in Vibhavadi District, Surat Thani Province, Southern Thailand. J Trop Med Parasitol.

[20] WHO (2016). Consolidated guidelines on the use of antiretroviral drugs for treating and preventing HIV infection Second edition ed.

[21] Kasirye RP, Baisley K, Munderi P, Levin J, Anywaine Z, Nunn A, Kamali A, Grosskurth H. Incidence of malaria by cotrimoxazole use in HIV-infected Ugandan adults on antiretroviral therapy: a randomised, placebo-controlled study. AIDS (London, England). 2016;30(4):635. 10.1097/QAD.0000000000000956 PMC4732005 26558729

[22] BedimoBeyenea H, MulualemTadesse HD, Beyenee MB (2017). Concurrent *Plasmodium* infection, anemia and their correlates among newly diagnosed people living with HIV/AIDS in Northern Ethiopia. Acta Trop.

[23] Alemayehu G, Melaku Z, Abreha T, Alemayehu B, Girma S, Tadesse Y, Gadisa T, Lulseged S, Balcha TT, Hoos D, Teka H. Burden of malaria among adult patients attending general medical outpatient department and HIV care and treatment clinics in Oromia, Ethiopia: a comparative cross-sectional study. Malaria J. 2015;14(1):501. 10.1186/s12936-015-1029-0 PMC4681039 26671012

[24] Organization WH (2014). March 2014 supplement to the 2013 consolidated guidelines on the use of antiretroviral drugs for treating and preventing HIV infection: recommendations for a public health approach.

[25] Kamya MR, Byakika-Kibwika P, Gasasira AF, Havlir D, Rosenthal PJ, Dorsey G, Achan J. The effect of HIV on malaria in the context of the current standard of care for HIV-infected populations in Africa. Future virology. 2012;7(7):699–708. 10.2217/FVL.12.59 PMC3535690 23293660

[26] Hassani AS, Marston BJ. Impact of cotrimoxazole and insecticide-treated nets for malaria prevention on key outcomes among HIV-infected adults in low-and middle-income countries: a systematic review. J Acquir Immune Defic Syndr. 2015;68(Suppl 3):S306. 10.1097/QAI.0000000000000522 PMC4818759 25768870

[27] Hassan ZI, Afolaranmi TO, Amaike C, Oyebode T, Gadzama DA, Zoakah AI. Effect of long lasting insecticide treated net on incidence of Malaria among people living with HIV/AIDS in Bassa Local Government Area of Plateau State, North Central Nigeria.

[28] Walson JL, Sangaré LR, Singa BO, Naulikha JM, Piper BK, Yuhas K (2013). Evaluation of impact of long-lasting insecticide-treated bed nets and point-of-use water filters on HIV-1 disease progression in Kenya. Aids..

[29] Agegnehu F, Shimeka A, Berihun F, Tamir M. Determinants of malaria infection in Dembia district, Northwest Ethiopia: a case-control study. BMC Publ Health. 2018;18(1):480. 10.1186/s12889-018-5370-4 PMC5896134 29642899

[30] Afolaranmi TO, Hassan ZI, Amaike C, Miner CA, Oyebode T. Effect of health education on knowledge of malaria and long lasting insecticide-treated nets among clients accessing care in the out-patient Department of a Secondary Health Facility in Plateau State, Nigeria. J Med Tropics. 2015;17(2):65. 10.4103/2276-7096.162283 PMC8186277 34109137

[31] Amoran OE. Impact of health education intervention on malaria prevention practices among nursing mothers in rural communities in Nigeria. Nigerian Medical Journal: Journal of the Nigeria Medical Association. 2013;54(2):115. 10.4103/0300-1652.110046 PMC3687863 23798798

[32] USID C, US. Presidents malaria initiative. In door residual spraying 2011. Available from: http://www.africairs.net/indoor-residual-spraying/.

[33] Gimnig JE, Otieno P, Were V, Marwanga D, Abong’o D, Wiegand R (2016). The effect of indoor residual spraying on the prevalence of malaria parasite infection, clinical malaria and anemia in an area of perennial transmission and moderate coverage of insecticide treated nets in Western Kenya.

[34] Skarbinski J, Mwandama D, Wolkon A, Luka M, Jafali J, Smith A, Mzilahowa T, Gimnig J, Campbell C, Chiphwanya J, Ali D. Impact of indoor residual spraying with lambda-cyhalothrin on malaria parasitemia and anemia prevalence among children less than five years of age in an area of intense, year-round transmission in Malawi. Am J Tropical Med Hygiene. 2012;86(6):997–1004. 10.4269/ajtmh.2012.11-0621 PMC3366547 22665608

